# AI-Powered Very-High-Cycle Fatigue Control: Optimizing Microstructural Design for Selective Laser Melted Ti-6Al-4V

**DOI:** 10.3390/ma18071472

**Published:** 2025-03-26

**Authors:** Mustafa Awd, Frank Walther

**Affiliations:** 1 Institute for Informatics and Automation (IIA), Bremen City University of Applied Sciences (HSB), Flughafenallee 10, D-28199 Bremen, Germany; 2 Testia GmbH, Airbus Group, Cornelius-Edzard-Straße 15, D-28199 Bremen, Germany; 3 Chair of Materials Test Engineering (WPT), Faculty of Mechanical Engineering, TU Dortmund University, Baroper Str. 303, D-44227 Dortmund, Germany; frank.walther@tu-dortmund.de

**Keywords:** machine learning, additive manufacturing, very high cycle fatigue (VHCF) resistance, microstructural optimization, process parameter optimization

## Abstract

Integrating machine learning into additive manufacturing offers transformative opportunities to optimize material properties and design high-performance, fatigue-resistant structures for critical applications in aerospace, biomedical, and structural engineering. This study explores mechanistic machine learning techniques to tailor microstructural features, leveraging data from ultrasonic fatigue tests where very high cycle fatigue properties were assessed up to $1\times 10^{10}$ cycles. Machine learning models predicted critical fatigue thresholds, optimized process parameters, and reduced design iteration cycles by over 50%, leading to faster production of safer, more durable components. By refining grain orientation and phase uniformity, fatigue crack propagation resistance improved by 20–30%, significantly enhancing fatigue life and reliability for mission-critical aerospace components, such as turbine blades and structural airframe parts, in an industry where failure is not an option. Additionally, the machine learning-driven design of metamaterials enabled structures with a 15% weight reduction and improved yield strength, demonstrating the feasibility of bioinspired geometries for lightweight applications in space exploration, medical implants, and high-performance automotive components. In the area of titanium and aluminum alloys, machine learning identified key process parameters such as temperature gradients and cooling rates, which govern microstructural evolution and enable fatigue-resistant designs tailored for high-stress environments in aircraft, biomedical prosthetics, and high-speed transportation. Combining theoretical insights and experimental validations, this research highlights the potential of machine learning to refine microstructural properties and establish intelligent, adaptive manufacturing systems, ensuring enhanced reliability, performance, and efficiency in cutting-edge engineering applications.

## 1. Introduction

Recent advances suggest that integrating machine learning (ML) into AM processes can address these challenges by predicting and enhancing fatigue properties. By integrating advanced sensor technologies and inline monitoring with machine learning algorithms, manufacturers can capture high-fidelity data on process parameters (e.g., laser power, scan speed, spot size, and hatch distance) and build layer characteristics (e.g., melt pool dimensions and thermal gradients) in real-time, enabling predictive models to detect anomalies and recommend parameter adjustments that improve microstructure uniformity and reduce fatigue-critical defects [1]. These models, often based on supervised or reinforcement learning approaches [2], are further refined by incorporating post-build inspection data, such as X-ray computed tomography or ultrasonic testing, to correlate internal defects with fatigue performance [3] and guide process optimization as well as targeted secondary exposures during in situ treatment steps or offline heat treatment [4]. By continuously retraining on new data, this ML-driven feedback loop substantially improves the reliability of AM parts, ensuring reproducible microstructural features and mitigating stress concentrations that can lead to premature fatigue failure. Moreover, metadata can be used to design microstructures with predetermined properties, and models can design the process parameters that could lead to the desired properties [5].

### 1.1. Machine Learning for Predicting Fatigue Properties in Additive Manufacturing

Additive manufacturing often results in components with variable fatigue properties due to factors like process parameters, microstructural inconsistencies, and residual stresses. Traditional methods of evaluating these properties are time-consuming and costly. Recent studies have applied ML models, such as artificial neural networks, support vector machines, and random forests, to predict fatigue life and strength based on AM process parameters and resulting microstructures. These models have shown promise in accurately forecasting fatigue performance, thereby reducing the need for extensive physical testing. Liu et al. presented an integrated experimental and computational framework for analyzing cold dwell fatigue in titanium alloys, focusing on microstructure-sensitive cold dwell debit. Their study underscored the importance of crystal-level slip properties and microstructural heterogeneity in predicting fatigue life [6]. Another approach to fatigue analysis involves ultrasonic frequency testing. Fitzka et al. examined the usability of ultrasonic frequency testing for the rapid generation of high and very high cycle fatigue data, comparing fatigue properties measured using ultrasonic and conventional equipment. Their findings emphasized the potential of high-frequency testing in significantly reducing testing times while maintaining accuracy [7]. In addition to testing methodologies, controlling grain structure and defects is crucial for enhancing fatigue properties. Mukherjee et al. reviewed strategies for optimizing grain structure, phase composition, and defect control in additively manufactured high-performance metallic components. Their paper highlighted the role of digital tools, including machine learning, in refining processing parameters and improving material properties [8]. By integrating computational models with experimental validation, they provide valuable insights into optimizing manufacturing processes and material design for improved fatigue resistance.

### 1.2. ML-Driven Microstructural Optimization in Titanium and Aluminum Alloys

ML algorithms are being utilized to predict and control microstructural features in titanium and aluminum alloys, aiming to achieve desired mechanical properties. For example, models have been developed to forecast microstructural evolution during processing, enabling the design of microstructures or complete new alloys with customized properties. These optimized alloys are particularly valuable in aerospace and biomedical sectors, where specific microstructural characteristics are critical for performance. ML-assisted design accelerates the development of alloys with enhanced strength-to-weight ratios and improved biocompatibility. Liu et al. examined crack initiation in micro-texture regions of Ti-6Al-4V during high-cycle fatigue tests, employing crystal plasticity modeling to understand fatigue mechanisms [9]. Meanwhile, Nguyen et al. critically reviewed additive manufacturing processes for Ti-6Al-4V, highlighting their impact on microstructure and mechanical properties [10]. Other studies investigated phase transformations, deformation behaviors, and microstructure refinement strategies in titanium alloys. For example, Hua et al. explored the interaction between phase transformation and deformation during hot compression in metastable β titanium alloys [11], while Davis et al. analyzed the effects of inter-pass deformation on microstructure refinement in additively manufactured wire-arc titanium [12]. Furthermore, Chen et al. studied stress-induced ω phase transformation and its implications for microstructural evolution and adiabatic shearing behavior [13], and Schuch et al. assessed the mechanical behavior of additively manufactured Ti6Al4V under varying loading conditions [14]. Worsnop et al. further contributed by evaluating the influence of alloy elements on slip intermittency and dwell fatigue in titanium [15].

Parallel advancements have been made in aluminum alloys, where machine learning techniques are increasingly being leveraged to predict and enhance fatigue resistance. Awd et al. applied machine learning models to forecast fatigue strength in hybrid and additively manufactured aluminum alloys within the very high cycle fatigue regime [16]. Sun et al. focused on residual stress analysis in additively manufactured wire arc aluminum components [17], while Böhm et al. developed a practical approach to mitigate solidification cracks during laser powder bed fusion [18]. Cheng et al. conducted nanoindentation studies on Al-8Ce-10Mg alloys, employing crystal plasticity finite element modeling to understand local mechanical responses [19]. Furthermore, Mason et al. proposed a strain rate-dependent plasticity model tailored for aerospace-grade aluminum alloys produced via solid-state additive manufacturing [20]. Lastly, Hu et al. investigated methods to inhibit weld cracking in high-strength aluminum alloys, which addresses a critical challenge in manufacturing [21] and integrates experimental and computational approaches, including machine learning, to optimize material properties and improve fatigue resistance in various engineering applications. In aerospace, the control of grain orientation and phase morphology in Ti-6Al-4V is critical for enhancing fatigue life in turbine blades and airframe components, where cyclic loading at high frequencies can lead to premature failure if microstructures are not optimized [9]. In biomedical implants, particularly for load-bearing components such as hip and spinal implants, microstructural homogeneity and pore distribution directly affect both osseointegration and long-term mechanical reliability [22]. For structural applications, such as high-speed rail components or bridges, optimizing grain boundary character distribution in aluminum alloys helps resist crack initiation and propagation under VHCF loading conditions [23].

### 1.3. ML-Enhanced Design of Fatigue-Resistant Metamaterials

ML facilitates the creation of metamaterials with enhanced fatigue resistance by optimizing complex geometries and material compositions. Deep learning models, combined with genetic algorithms, have been employed to inversely design microstructures that meet specific mechanical property requirements. Recent advancements in machine learning (ML) have significantly contributed to the design and prediction of fatigue-resistant metamaterials. Farid [24] introduced a hybrid model combining artificial neural networks and Gaussian process regression to predict fatigue failure under stochastic loading. This model not only predicts the time to failure but also quantifies uncertainty, providing real-time decision-making capabilities for structural health monitoring. Addressing the limitations of traditional extreme value statistics in fatigue strength prediction, Minerva et al. [3] applied supervised ML algorithms to improve the classification of defects in Laser-Powder Bed Fusion components, leading to more robust fatigue strength predictions. Additionally, Salvati et al. [25] developed a physics-informed neural network framework that integrates fracture mechanics constraints, which enhanced fatigue life prediction accuracy by considering the real morphology of defects. Maleki et al. [26] explored the efficiency of ML in assessing fatigue performance in post-processed additively manufactured AlSi10Mg, though the abstract does not provide specific details on their findings. Similarly, Awd et al. [5] investigated the fatigue strength of hybrid and additively manufactured aluminum alloys in the very high cycle fatigue (VHCF) regime by using ML techniques, although their methodology and results are not detailed in the abstract. By integrating advanced computational approaches with experimental data, ML-driven models offer new insights and methodologies that can significantly improve material design and fatigue performance prediction in additive manufacturing.

### 1.4. Integration of ML in Additive Manufacturing for Process Optimization

ML models are applied to identify optimal AM process parameters, such as laser power, scanning speed, and layer thickness, which influence the fatigue properties of manufactured components. By analyzing large datasets, these models can predict the outcomes of different parameter settings, leading to improved component performance. Incorporating ML into the AM workflow aids in real-time monitoring and detection of defects that could compromise fatigue resistance. Advanced algorithms analyze sensor data to identify anomalies during the manufacturing process, which enables immediate corrective actions. The integration of machine learning (ML) in additive manufacturing (AM) for process optimization is a burgeoning field, as highlighted by recent studies. Sahar et al. [27] provide a comprehensive review of ML applications in detecting anomalies during the laser powder bed fusion (L-PBF) process, a key technique in metal additive manufacturing. Their study emphasizes the potential of ML to enhance real-time process control, thus optimizing the manufacturing process for higher accuracy, reduced production time, and minimized material waste. While ML has the capability to significantly reform the AM process, the authors note that research in anomaly detection using ML remains limited and requires further exploration. Another significant application of ML in AM pertains to fatigue assessment. Maleki et al. [26] explore the efficiency of ML in evaluating the fatigue performance of post-processed additively manufactured AlSi10Mg. Although their abstract does not provide detailed findings, the study likely investigates how ML can be leveraged to predict and enhance fatigue performance. Salvati et al. [25] introduce a novel physics-informed neural network framework for predicting the fatigue life of defective materials in AM. This approach integrates fracture mechanics constraints into the ML model, allowing for more accurate fatigue life prediction by considering the real morphology of defects. By adhering to fundamental fracture mechanics laws and requiring fewer experimental data points, this framework offers a promising avenue for structural design with enhanced accuracy. Another application of ML in AM involves fatigue strength prediction. Minerva et al. [3] apply ML-assisted extreme value statistics to predict the fatigue strength of AlSi10Mg manufactured using L-PBF. Their study demonstrates that ML improves the robustness of fatigue strength predictions by classifying defects before estimating maxima distributions, leading to more accurate assessments of material performance. Wang et al. [28] extend the application of ML to high-entropy alloys by introducing a dimensionally augmented and physics-informed ML model for quality prediction in additively manufactured components. Their model connects process parameters with quality characteristics, demonstrating improved prediction accuracy and generalization capabilities for complex alloy systems. With respect to ML in complex systems, Falegnami et al. presented the interplay between Simplicity and Complexity by developing conceptual artifacts through a Design Science Research approach to aid in managing complex adaptive systems. The research distills key aspects of Simplexity across disciplines and proposes a shared framework for fields such as cyber-socio-technical system design, resilience engineering, and biological network analysis [29]. These studies collectively underscore the transformative potential of ML in optimizing additive manufacturing processes, particularly in enhancing accuracy, efficiency, and material performance.

In this study, machine learning (ML) techniques will be integrated into additive manufacturing (AM) to optimize material properties and enhance the design of fatigue-resistant structures. A key focus will be the development of mechanistic ML models to tailor microstructural features and predict critical fatigue thresholds. Data from ultrasonic fatigue tests, particularly in the very high cycle fatigue (VHCF) regime, will be utilized to improve the accuracy of fatigue life predictions. The study aims to refine grain orientation and phase uniformity, leading to a 20–30% increase in fatigue crack propagation resistance and significantly enhancing overall fatigue life.

Additionally, this research will explore the optimization of process parameters, such as temperature gradients and cooling rates, for governing microstructural evolution in titanium and aluminum alloys. By leveraging ML-driven approaches, the study will identify optimal conditions to reduce design iteration cycles by over 50%, thus improving manufacturing efficiency and structural performance. A physics-informed ML framework will be developed to integrate fracture mechanics constraints into predictive models, minimizing experimental requirements while enhancing accuracy.

Furthermore, the ML-driven design of metamaterials will be investigated to enable the creation of lightweight, high-performance structures. This study will evaluate how bioinspired geometries, optimized through ML algorithms, can lead to up to a 15% weight reduction while maintaining or improving yield strength. Real-time process monitoring and anomaly detection using ML techniques will also be implemented to enhance manufacturing precision, reduce material waste, and improve structural integrity.

By combining theoretical insights with experimental validation, this research will establish an intelligent, adaptive manufacturing framework. The outcomes of this study will demonstrate how ML can refine microstructural properties, improve fatigue performance, and optimize AM processes for aerospace, biomedical, and structural applications.

## 2. Materials and Methods

### 2.1. Process Window

In this study, we will introduce the process window used to train the machine learning (ML) model for optimizing fatigue resistance in additively manufactured structures. The dataset consists of specimens of Ti-6Al-4V produced by selective laser melting (SLM) in the vertical direction. One batch will be fabricated using optimized process parameters with a single laser exposure, while subsequent batches will incorporate a secondary exposure with controlled variations. These variations are expected to induce microstructural differences and defect distributions, enabling a comprehensive analysis of their effects on fatigue behavior.

The collected data, which includes fatigue model predictions and distributions of key microstructural features, will be used to train and validate the ML model. The predictive framework will be applied in Section 2.2 and Section 2.3 to assess its performance across different modeling techniques. Additionally, this section will introduce the concept of mechanical energy dissipation through atomic damping as a microstructural factor influencing fatigue resistance. This concept will later be explored in the context of physics-informed machine learning (PIML) in Section 2.3.

The process window defining laser scanning parameters is presented in Table 1, which explains how the control of the microstructure is attained during SLM by implementing adaptations of the scanning parameters in the second exposure. Here, Ev represents energy density, *P* denotes laser power, vs corresponds to scanning speed, and *D* indicates spot size. The specimens were fabricated using modified SLM 250 HL and SLM 500 HL systems for AlSi10Mg and Ti-6Al-4V alloys, respectively. All specimens were built at a 90° orientation relative to the building platform. To introduce controlled thermal history effects, a second exposure treatment was applied at varying intensities, influencing the cooling rates and microstructural evolution of the material.

**Figure 1:**
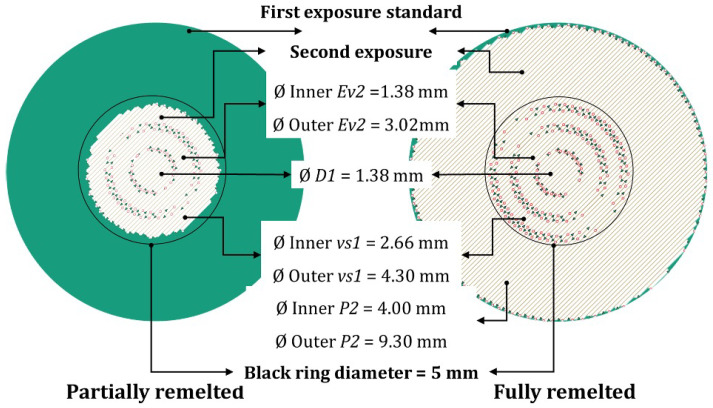
Hatching strategy including the different process parameters for secondary exposures according to Table 1.

More details regarding the manufacturing technique can be found in [1]. Figure 1 schematically illustrates the functional grading strategy implemented in this study, where already exposed layers are selectively remelted. It explains how the control of the microstructure is attained during SLM by implementing adaptations of the scanning parameters in the second exposure. A first exposure is carried out using the standard parameter dataset in Table 1. Then, it is possible for a second exposure to be applied at various depths of the cross section according to the distribution in Figure 1 with the corresponding scanning parameters from Table 1. This strategy aims to refine local properties by applying a mechanistic learning algorithm to optimize fatigue resistance and microstructural stability.

### 2.2. Very High Cycle Fatigue (VHCF) and Ultrasonic Fatigue Testing (USF)

Resonance occurs in spring when the frequency of the applied force is comparable to the characteristic vibration of the spring. The resonance phenomenon arises when the frequency of the excitation force in an oscillating system matches the system’s natural frequency. When there is no loss of energy, resonance will take place.

The energy of the oscillator can be expressed as follows [30]:

(1) $$E\left(R\right)=\frac{1}{2}\mu \nu^{2}+\frac{1}{2}\kappa R^{2}$$

where μ is the reduced mass, ν is the velocity, and κ is the stiffness constant. The stiffness constant can be determined through the second derivative of the energy with respect to *R*:

(2) $$\kappa =\frac{d^{2}E}{dR^{2}}.$$

The internal damping (*ID*) is determined through the following relation:

(3) $$ID=\frac{1}{2\pi }\frac{W_{\mathrm{diss}}}{W_{\mathrm{el}}}$$

where $W_{\mathrm{diss}}$ is the amount of energy lost in a given volume unit throughout one vibration cycle and $W_{\mathrm{el}}$ is the maximum amount of elastic energy stored in a given volume [31].

(4) $$W_{\mathrm{diss}}=\oint \sigma \cdot d\varepsilon =\pi \sigma_{0}\varepsilon_{0}\sin\phi =\pi J_{2}\sigma_{0}^{2}$$

(5) $$W_{\mathrm{el}}=\int_{0}^{\sigma_{0}}\sigma \cdot d\varepsilon =\frac{1}{2}J_{1}\sigma_{0}^{2}$$

where $J_{1}$ and $J_{2}$ are the real and imaginary parts of the compliance $J^{*}$. Hence, the elastic modulus *E*, as an intensive stiffness parameter and the inverse of compliance, plays a critical role in the elastic energy stored in the fatigue specimen being tested at an ultrasonic resonance testing device. It should come as no surprise, therefore, that

(6) ID=tanϕ

The displacement amplitude *x* at the end of the fatigue specimen is governed by the harmonic equation of motion [32]:

(7) $$x\left(t\right)=x_{m}e^{-\beta t}\cos\left(t\sqrt{\omega^{2}-\beta^{2}}+\Phi \right)+X_{m}\cos\left(\omega_{w}t+\phi \right)$$

where ω is the angular frequency, $\omega_{w}$ is the excitation frequency, and β is the damping ratio. The amplitude reaches its maximum at resonance frequencies. The role of elastic properties highlights their significance in constructing an accurate loading case for a fatigue specimen subjected to ultrasonic mechanical resonance. The larger the imaginary part $J_{2}$ of the overall compliance $J^{*}$, the less adequate the specimen design based solely on elastic properties. Therefore, these characteristics must be well-defined as critical boundary conditions for the testing process.

Under a specific stress threshold, it was previously believed that fatigue strength is not affected. And this limit is material-dependent, in such a way that fatigue fracture originates from microscopic heterogeneities, provided that fatigue loading under this threshold is applied for a sufficiently long time. Hence, the effective fatigue damage resistance mechanisms change. The primary regime of the effect of such heterogeneity becomes a research field of very high cycle fatigue damage mechanisms made possible only by the introduction of high-frequency fatigue testing systems. Such a study is only possible with the generation of proper fatigue data beyond 1E8 cycles. Generation of such data is economically prohibitive using conventional low-frequency systems. The advent of these systems allowed researchers to rename the previously identified fatigue limit as the limit of fatigue damage mechanisms transfer from mesoscopic heterogeneities to microscopic heterogeneities. Only ultrasonic fatigue testing systems that reach 1E9 cycles in 14 h at 20 kHz continuous loading mode are economically feasible to go deep into this territory.

**Figure 2:**
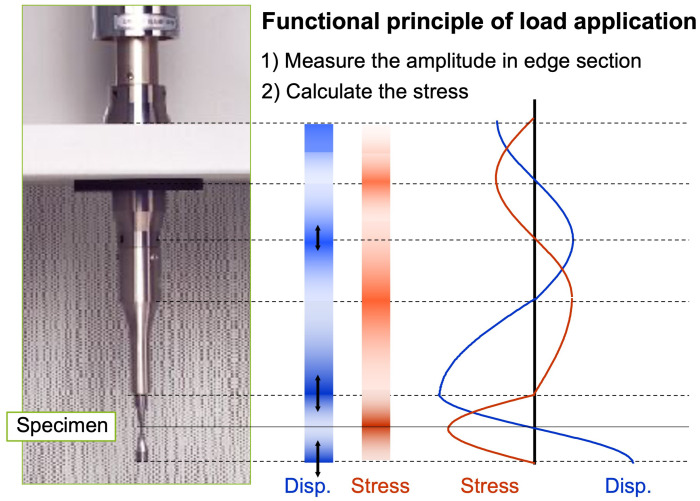
Stress and displacement profile during ultrasonic loading at USF-2000A.

The system itself is simply structured, with an essential component being an ultrasonic pulse generator. As with all elastic waves, ultrasonic waves may vary according to the nature and size of the body through which they propagate [33]. Therefore, practically, any mechanical device capable of vibrating within the ultrasonic frequency range can serve as an ultrasound source; see Figure 2.

In general, systems that produce ultrasonic waves must distinguish between the system that provides energy (piezoelectric actuator) and the element that receives this energy (booster and horn) and transfer it to another system (the very high cycle fatigue specimen) in the form of high-frequency mechanical vibration. This component belongs to the class of devices known as transducers. At mechanical resonance, the transducer operates as a resistance-based electric load, either $R_{e}^{\prime }$ or $R_{e}^{\prime \prime }$, depending on the specific case, alongside the electrostatic capacitance $C_{0}$ of the quartz plate, which is arranged in parallel. The reactance of $C_{0}$ can be canceled using a coil of inductance

(8) $$L_{0}=\frac{1}{4\pi^{2}f_{0}^{2}C_{0}}$$

where $f_{0}$ is the resonance frequency of the crystal. The coil $L_{0}$ has movable connections that allow the user to determine the optimal operating conditions. X-cut quartz plates are suitable for the generation of longitudinal or extensional waves. The so-called electromechanical coupling factor k is often a factor that must be considered when analyzing a piezoelectric transducer. It is defined as the square root of the ratio of the energy stored in the mechanical form to the total electrical energy provided to the crystal for a specific kind of strain. A piezoelectric crystal is employed in the ultrasonic fatigue testing systems in the very high cycle fatigue regime. The elastic ultrasonic waves, which are generated, are amplified into a microscale displacement in the booster and transferred to the specimen through the horn. The maximum displacement amplitude at the end of the horn depends on the physical and geometric properties of the booster and horn in such a way that Equation (6) has to be satisfied, and energy must be conserved. An overview of such a system can be seen in Figure 2, which is compliant with the standard of the Japanese Welding Society for very high cycle fatigue testing [34].

**Figure 3:**
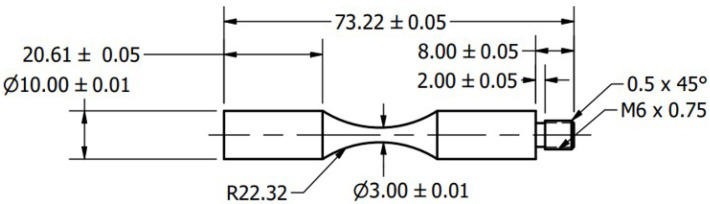
Specimen design in mm of a Ti-6Al-4V specimen at USF-2000A.

Since the specimen should be vibrating in the longitudinal direction, the natural frequency of the elastic oscillation determines the maximum elongation of the free ends in the two opposite directions, characterized by a standing elastic wave with displacement antinodes on both faces [33]. This mechanism is responsible for establishing the load case in the middle of the specimen. Figure 3 shows an axially loaded hourglass-shaped specimen. In the case of an axially loaded fatigue specimen, the axial stress is given by the following:

(9) $$\sigma_{a}=A_{0}E\beta \frac{\cos\left(kL_{2}\right)\cosh\left(\alpha L_{1}\right)}{\sinh(\beta L_{1})}$$

where

(10) $$k=2\pi f\sqrt{\frac{\rho }{E}}$$

(11) $$\alpha =\frac{1}{L_{1}}\cosh^{-1}\left(\frac{D_{2}}{D_{1}}\right)$$

(12) $$\beta =\sqrt{\alpha^{2}-k^{2}}$$

where *f* is the resonance frequency, *E* is the modulus of elasticity, ρ is the density, and $L_{1},\;L_{2},\;D_{1},$ and $D_{2}$ are specimen dimensions defined in Figure 3, where 8 specimens were tested for every configuration.

Therefore, a well-designed specimen should avoid the entertainment phenomenon. Countermeasures should be incorporated into the experimental setup, such as a user warning or a frequency check feature. When a sufficiently large periodic force (i.e., large *F*) with a frequency ω close to the natural frequency of the limit cycle is applied, the specimen can abandon its natural oscillation and vibrate at the frequency of the applied force. This is known as the entertainment phenomenon of the very high cycle fatigue specimen. The risk associated with this phenomenon is that, although the specimen may oscillate at the forced frequency of the system, the load case will not be accurate. In the case of torsional loading, the shear stress is given by

(13) $$\tau_{a}=A_{0}G\beta \frac{\cos\left(kL_{2}\right)\cosh\left(\alpha L_{1}\right)}{\sinh(\beta L_{1})}$$

where

(14) $$k=2\pi f\sqrt{\frac{\rho }{G}}$$

(15) $$\alpha =\frac{1}{L_{1}}\cosh^{-1}\left(\frac{D_{2}^{2}}{D_{1}^{2}}\right)$$

(16) $$\beta =\sqrt{\alpha^{2}-k^{2}}$$

where *G* is the modulus of transverse elasticity. To summarize, both the system design and the specimen must ensure free vibration and avoid forced vibration. The horn must be able to compensate only for damping, which will increase over time due to the damaged state of the fatigue specimen. This change in damping can also be used as a measure of damage accumulation. A detailed discussion on the difference between forced and free vibration can be found in [35].

Hence, to ensure proper specimen design, the eigenvalue motion problem in Equation (6) must be solved either analytically in simple cases or numerically in more complex specimen designs with non-trivial setups. Above all, the natural frequency of the specimen must not be significantly lower than the oscillation excitation frequencies of the actuator, booster, and horn to establish resonance. In the case of non-trivial specimen geometries or arbitrary contours, finite element analysis (FEA) is the most versatile numerical solution method in the frequency extraction procedure. This method performs the following:

- It performs eigenvalue extraction to calculate the natural frequencies and the corresponding mode shapes of a system.
- It accounts for initial stress and load stiffness effects due to preloads and initial conditions if geometric nonlinearity is included in the base state, allowing small vibrations of a preloaded structure to be modeled.

The eigenvalue problem for the natural frequencies of an undamped finite element model is formulated as follows [35]

(17) $$\left(-\omega^{2}M^{MN}+K^{MN}\right)\phi^{N}=0$$

where $M^{MN}$ is the mass matrix (which is symmetric and positive definite), $K^{MN}$ is the stiffness matrix (which includes initial stiffness effects if the base state accounts for nonlinear geometry), and $\phi^{N}$ is the eigenvector representing the mode of vibration.

This solution must satisfy the principle of minimum potential energy for linear elastic bodies with zero membrane stresses [36], which is expressed as follows:

(18) $$L_{1}\left(\alpha \right)=\int_{\tau }\left(\frac{1}{2}C^{ijkl}\varepsilon_{ij}\varepsilon_{kl}-\tilde{f}\cdot \tilde{w}\right)d\tau -\int_{\sigma }\tilde{p}\cdot \tilde{w}d\sigma +O\left(\alpha \right)=\min$$

where *w* is the displacement field of a corresponding amplitude $w_{A}$ for a given circular frequency, ω, at which the specimen vibrates:

(19) $$w=w_{A}\sin\left(\omega t\right)$$

The strain and kinetic energy of the specimen are discretized in the finite element model as follows:

(20) $$E_{p}^{e}=\frac{1}{2}{\left\{w_{A}^{e}\right\}}^{T}\left[k^{e}\right]\left\{w_{A}^{e}\right\}\sin^{2}\left(\omega t\right)$$

(21) $$E_{k}^{e}=\frac{1}{2}{\left\{w_{A}^{e}\right\}}^{T}\left[m^{e}\right]\left\{w_{A}^{e}\right\}\omega^{2}\cos^{2}\left(\omega t\right)$$

where $\left\{w_{A}^{e}\right\}$ is the displacement amplitude vector of the element, $E_{p}^{e}$ and $E_{k}^{e}$ represent the strain and kinetic energy of the element, respectively, and $\left[k^{e}\right]$ and $\left[m^{e}\right]$ are the stiffness matrix and mass matrix of the element, respectively [37]. These matrices are conventional finite element matrices.

During free vibration, the total energy is preserved, confirming resonance, as highlighted earlier. In the middle of the specimen, the strain energy reaches its maximum, while the kinetic energy is zero. This condition leads to the following expression:

(22) $$E^{e}=\frac{1}{2}{\left\{w_{A}^{e}\right\}}^{T}\left[k^{e}\right]\left\{w_{A}^{e}\right\}+\Gamma$$

where $E^{e}$ is the total energy of an element in the finite element discretized system of a membrane, and Γ represents the work performed by the nodal force vector of the element when the system reaches its maximum energy. Microscopic features that may induce hardening and low thermal conductivity will lead to heat accumulation within the testing specimen. The temperature change beyond 10–15 K will validate the test result and lead to incomprehensible Woehler curves. If the damping gets significantly high, resonance will cease. Therefore, it is essential to keep specimen temperature within acceptable limits by either intermittent driving (pulse-pause loading mode) or a cooling medium. Intermittent driving implies that the specimen is excited by the actuator through the booster-horn couple for a period and then allowed to vibrate freely. The specimen dampens enough to dissipate heat to the surrounding cooling medium, which should have non-surface-active constituents that can influence the test result. The period of free vibration and damping is material-dependent on the rate of hardening, temperature generation, and thermal conductivity, which influences the heat dissipation rate.

### 2.3. Model Build-Up and Training

Simulating fatigue behavior is a computationally intensive task due to its dynamic complexity. The conventional cycle-by-cycle implicit time integration approach is often impractical due to its high computational demands. To overcome this, the present study adopts a methodology that maps the time-dependent displacement field onto the frequency domain. In the physics-informed ML model will impose a boundary and initial value conditions as terms in the neural network. This transformation is achieved using a truncated Fourier series (TFS) within a Newton-Raphson iterative scheme until a predefined residual threshold is attained.

By employing a truncated Fourier series, the displacement boundary conditions and their associated residuals are defined as follows [38]

(23) $$\bar{u}\left(t\right)=u_{0}+\sum_{k=1}^{n}\left[u_{k}^{s}\sin\left(k\omega t\right)+u_{k}^{c}\cos\left(k\omega t\right)\right]$$

(24) $$\bar{R}\left(t\right)=R_{0}+\sum_{k=1}^{n}\left[R_{k}^{s}\sin\left(k\omega t\right)+R_{k}^{c}\cos\left(k\omega t\right)\right]$$

where *n* represents the number of terms in the Fourier series, ω=2π/T is the angular frequency, and $u_{0}$, $u_{k}^{s}$, and $u_{k}^{c}$ denote the displacement coefficients corresponding to each degree of freedom in the system. The residual coefficients in Equation (24) are determined by evaluating the load response across the entire loading cycle. The Fourier series estimation based on the Newton Raphson scheme was implemented in Abaqus finite element software (https://www.3ds.com/products/simulia/abaqus) using a direct cyclic analysis that does not compute the damage state on a cycle-by-cycle basis but by extrapolating the damage using the displacement-frequency domain mapping in Equation (23).

To quantify energy dissipation during cyclic loading due to atomic damping of vibration energy, we implement a simplified form of the energy dissipation equation [39]

(25) $$dW_{damage}=\int_{0}^{\Delta \varepsilon }\sigma \;d\varepsilon$$

where σ represents the applied load, and Δε corresponds to the strain accumulation induced by cyclic loading. Part of this energy is irrecoverable, remaining as the residual work after accounting for heat dissipation and recoverable mechanical work

(26) $$dW_{irrecoverable}=dW_{supplied}-\left(dQ+dW_{recoverable}\right)$$

The irrecoverable work can be further decomposed into contributions from inelastic deformation, heat dissipation, and damage evolution, such that the damage component is proportionally related to both the irrecoverable and supplied work:

(27) $$dW_{irrecoverable}=dW_{inelastic}-\left(dQ+dW_{damage}\right)$$

(28) $$dW_{damage}\propto dW_{irrecoverable}$$

(29) $$dW_{damage}\propto dW_{supplied}$$

Fatigue failure occurs when the potential energy of a specimen is fully expended, and the specimen cannot work anymore to accommodate the loading and absorb the mechanical energy imposed, leading to the formation of new fracture surfaces while preserving the crystal lattice’s electronic structure. Since surface atoms exhibit higher energy than bulk atoms, the fatigue lifetime of a structure can be estimated as the ratio of potential energy to the damage per fatigue cycle, as suggested by Ellyin [39]

(30) $$\mathrm{Life}=\frac{\mathrm{Potential}\;\mathrm{energy}}{\mathrm{Damage}\;\mathrm{per}\;\mathrm{cycle}}=\frac{U_{0}}{dW_{damage}}$$

The simulations were carried out using the finite element software Abaqus. The state variables of the nodes were exported to be applied within Equation (37) in the Monte Carlo process to estimate the maximum aposteriori distribution of the fatigue strength. Therefore, the influences of stress concentration, shape factor, and certainty factor are taken into account at the defect scale and the macro scale of the specimen. In our fatigue forward problem, all the parameters, including the boundary conditions, are known. We will solve it as a pure partial differential equation (PDE) solving problem, in which the physics-informed neural network (PINN) is used as a traditional numerical method. The incomplete information that must complemented from sample data will be generated from the simulation according to Equation (30). Awd et al. showed this in detail in [5]. Let the unknown function u(x) be approximated by an ansatz u(x;w), which consists of a deep neural network with inputs $x=(x_{1},\ldots ,x_{d})$ and network weights *w*. The network has a single output, as *u* is a scalar function. In the case of a partial differential equation (PDE) system, there would be multiple outputs, one for each component. The ansatz is substituted into Equations (31) and (33) [40], and the objective is to determine the network weights *w* so that

(31) $$F\left(u\left(x;w\right),x,Du\left(x;w\right),D^{2}u\left(x;w\right),\ldots ,D^{k}u\left(x;w\right);\lambda \right)=f\left(x\right),\;x\in \Omega ,$$

(32) $$G\left(u\left(x;w\right),x,Du\left(x;w\right),D^{2}u\left(x;w\right),\ldots ,D^{m}u\left(x;w\right)\right)=g\left(x\right),\;x\in \Gamma .$$

The functions *F* and *G* in Equation (31) play distinct roles in enforcing the conditions of the problem. The function *F* enforces the PDE constraint by depending on the neural network approximation u(x;w) and its derivatives up to order *k*. It represents the residual of the governing partial differential equation (PDE), ensuring that the network solution satisfies the PDE inside the domain Ω. The objective is to find weights w such that F=f, where f(x) represents a known source term or function.

On the other hand, the function *G* enforces the boundary condition by involving the neural network approximation u(x;w) and its derivatives up to order *m*. It represents the residual of the boundary condition on Γ, the boundary of Ω, ensuring that the neural network solution satisfies the prescribed boundary conditions by making G=g, where g(x) is a known boundary function.

The key difference between the two functions is that *F* is associated with enforcing the PDE constraint within the domain Ω, whereas *G* ensures that the boundary conditions are satisfied on the boundary Γ. In the loss functions defined in Equation (31), $L_{r}\left(w\right)$ minimizes the PDE residual, while $L_{b}\left(w\right)$ minimizes the boundary residual. A valid solution for u(x;w) is achieved when both loss terms approach zero.

The partial derivatives $D^{\alpha }u\left(x;w\right)$ of the neural network output can be computed accurately and efficiently for given values of *x* and *w* using automatic differentiation techniques. To quantify the accuracy of the neural network approximation, we define the following loss functions:

(33) $$L_{r}\left(w\right)=\int_{\Omega }{\left|F\left(u\left(x;w\right),x,Du\left(x;w\right),D^{2}u\left(x;w\right),\ldots ,D^{k}u\left(x;w\right);\lambda \right)-f\left(x\right)\right|}^{2}dx,$$

(34) $$L_{b}\left(w\right)=\int_{\Gamma }{\left|G\left(u\left(x;w\right),x,Du\left(x;w\right),D^{2}u\left(x;w\right),\ldots ,D^{m}u\left(x;w\right)\right)-g\left(x\right)\right|}^{2}dx.$$

Since both $L_{r}\left(w\right)$ and $L_{b}\left(w\right)$ are non-negative, it follows that Equation (31) is satisfied, ensuring that u(x;w) adheres to the governing PDE and the boundary conditions throughout the domain (except on a set of measure zero) if and only if $L_{r}\left(w\right)=L_{b}\left(w\right)=0$. This is equivalently expressed as

(35) $$L_{r}\left(w\right)+L_{b}\left(w\right)=0$$

which numerically estimate Equation (31) by the finite element method for the domain variables Equation (6) and the Newton-Raphson scheme for the time-frequency domain Equation (23). Further details on the probabilistic regression function used to impose multivariate fatigue lifetime dependency on microstructural heterogeneity using Weibull regression can be found in [1,2,5]. The training followed an 80–20% split. In addition, an aposteriori maximization was implemented according to the Metropolis–Hastings algorithm and according to the acceptance ratio

(36) $$r=\frac{p(\theta^{*}|y)}{p(\theta^{\left(s\right)}|y)}=\frac{p\left(y|\theta^{*}\right)p\left(\theta^{*}\right)}{p\left(y|\theta^{\left(s\right)}\right)p\left(\theta^{\left(s\right)}\right)}$$

where we have a sampling model of the property given micro-structural heterogeneity Y∼p(y|θ) and a prior distribution p(θ) of that heterogeneity. Although in most problems, p(y|θ) and p(θ) can be evaluated for any given values of *y* and θ, the posterior distribution

(37) $$p\left(\theta |y\right)=\frac{p(\theta )p(y|\theta )}{\int p\left(\theta^{\prime }\right)p\left(y|\theta^{\prime }\right)\;d\theta^{\prime }}$$

is often difficult to compute due to the integral in the denominator [41]. Therefore, we implement the maximum aposteriori maximization according to [42].

## 3. Results and Discussion

### 3.1. Microstructural Heterogeneity for Strength Variability

**Figure 4:**
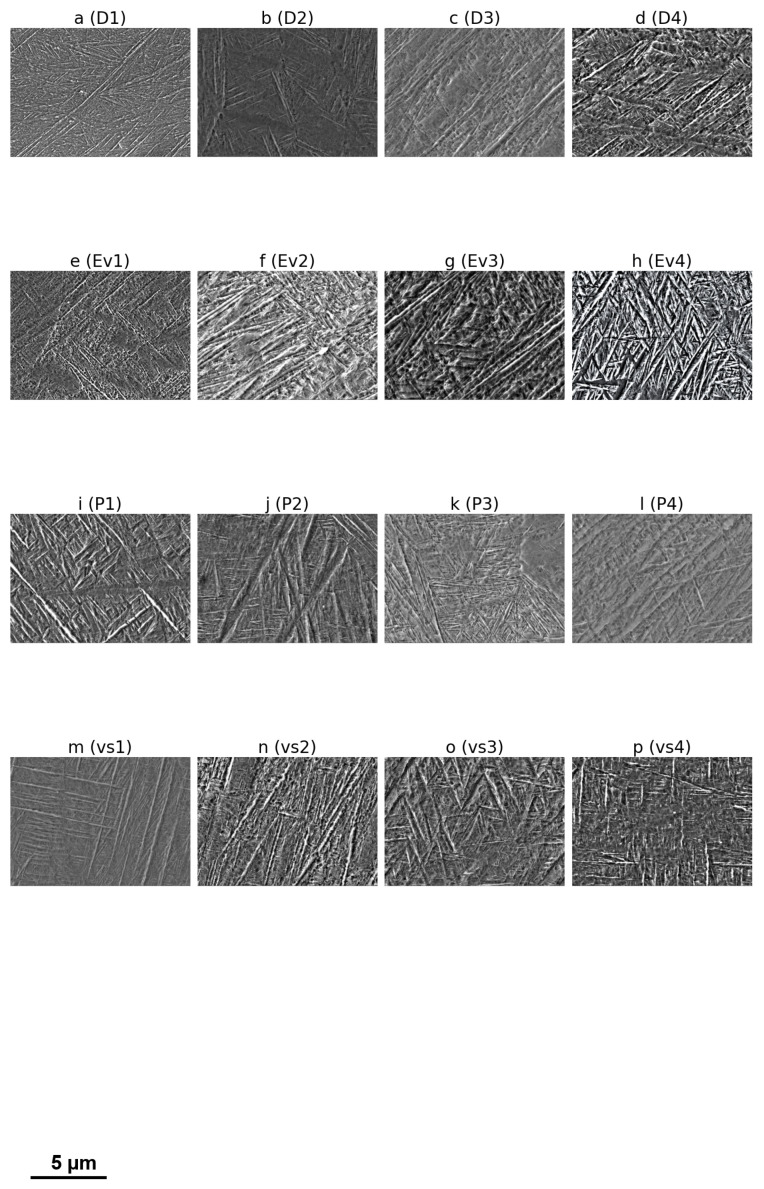
Dependency of microstructure heterogeneity on process parameters.

The model introduced in Section 2.3 relies on establishing a Weibull regression for the fatigue strength where the constraint and initial boundary conditions are enforced through a common loss function Equation (35). The Weibull regression parameters are estimated through sampling from a prior micro-structural distribution. The prior is difficult to estimate according to [42]; therefore, the maximum aposteriori estimation is implemented. Therefore, when we look at Figure 4, we can see an overview at high magnification of the batches introduced in Table 1. The variation in the width and length of the martensitic $\alpha^{\prime }$ phase and the prior β phase is influenced by the parameter set used in the secondary exposure, which alters the imposed cooling rate. However, in batches that saw a variation of spot size *D* and energy density $E_{v}$, the influence on the microstructure was minimal, see Figure 4. The model setup in Section 2.3 considers the physical aspects influencing the microstructure by imposing the transient and boundary conditions in Equations (31) and (33) on the Weibull regression process approximated by Monte Carlo process Equation (37). In comparison, during variation laser power *P* and scanning velocity $v_{s}$, significant coarsening of microstructural features was observed as total energy input led to a reduced cooling rate. Xiang et al. [43] investigated how the β (BCC) to α (HCP) martensitic transformation in Ti-6Al-4V alloy gives rise to diverse microstructural morphologies, with particular emphasis on the formation of habit planes and twin interfaces. By combining phenomenological martensitic theory (PTMT) and phase-field simulations, they showed that a near-334 habit plane emerges at the β/α interface, largely driven by lattice-parameter mismatches. Certain twin types (compound, Type I, and Type II) arise in the α phase to minimize elastic strain energy but often exhibit very small volume fractions, which is consistent with experimental reports that twins can be difficult to detect. These twins play a critical role in the crystal plastification that leads to microstructural crack formation. Awd et al. [16] explore the fracture behavior of SLM Ti-6Al-4V in the VHCF regime, though specific details of the study are not provided in the abstract. Fitzka et al. [7] and Avateffazeli [44] review ultrasonic fatigue testing as a method for rapid VHCF data generation, noting that while some materials show frequency sensitivity, titanium alloys like Ti-6Al-4V generally do not, making ultrasonic testing a viable approach. Nguyen et al. [10] provide a critical review of the microstructure and mechanical properties of Ti-6Al-4V produced by SLM, noting that while SLM can yield parts with superior strength, factors such as residual stress and porosity can affect fatigue life. Additionally, Cao et al. [45] investigate the fatigue response of AlSi10Mg, another SLM-produced alloy, focusing on the role of pores in fatigue crack nucleation and finding that lack of fusion pores significantly reduces fatigue life compared to gas/keyhole pores.

### 3.2. Model Predictions in VHCF for Different Microstructural Populations

**Figure 5:**
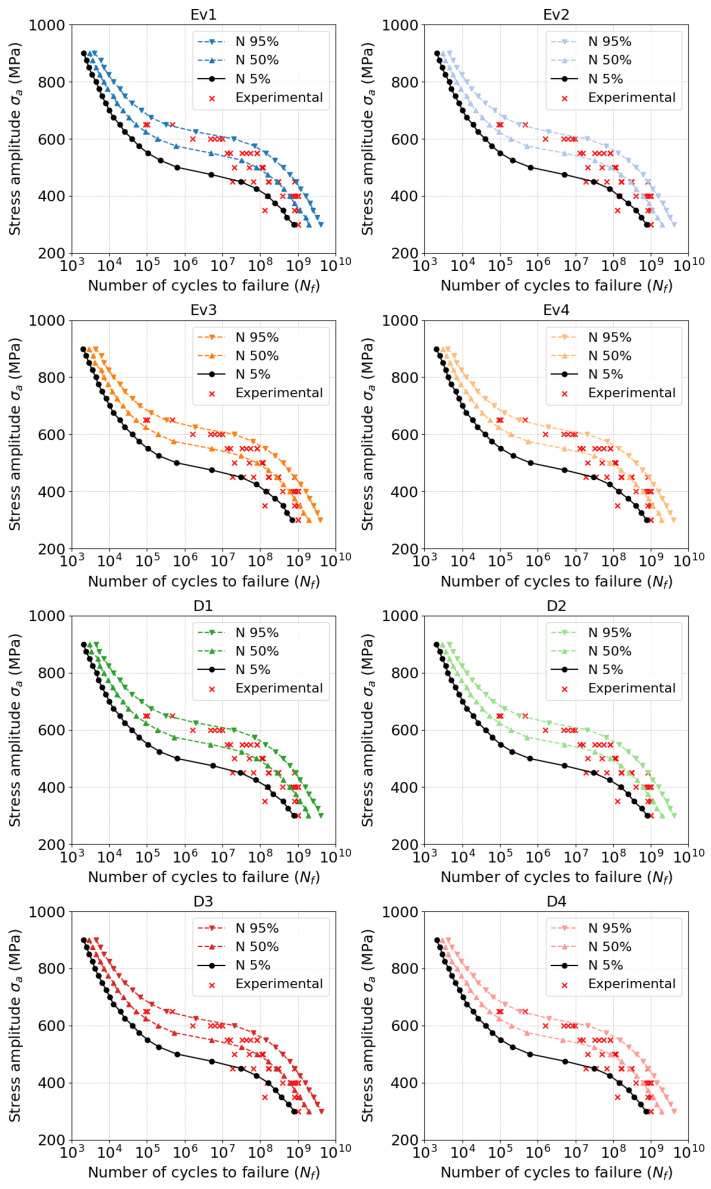
Predicted Woheler curves compared with experiments: Batches Ev1...D4.

**Figure 6:**
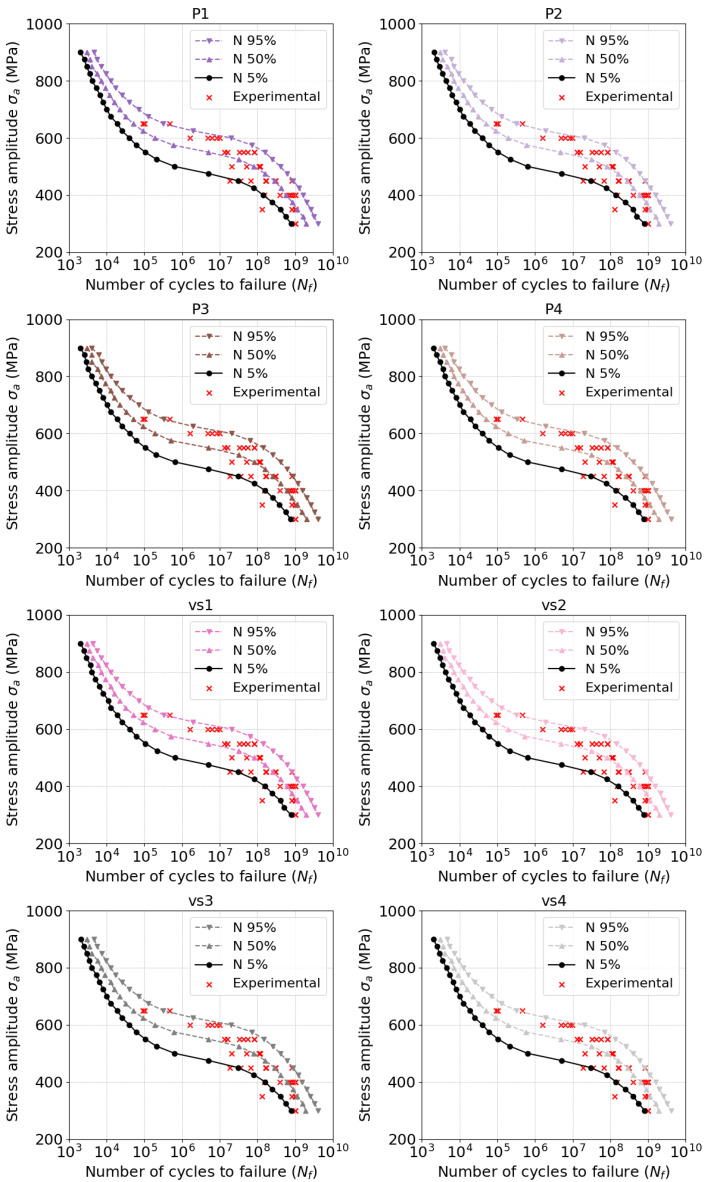
Predicted Woheler curves compared with experiments: Batches P1...vs4.

**Figure 7:**
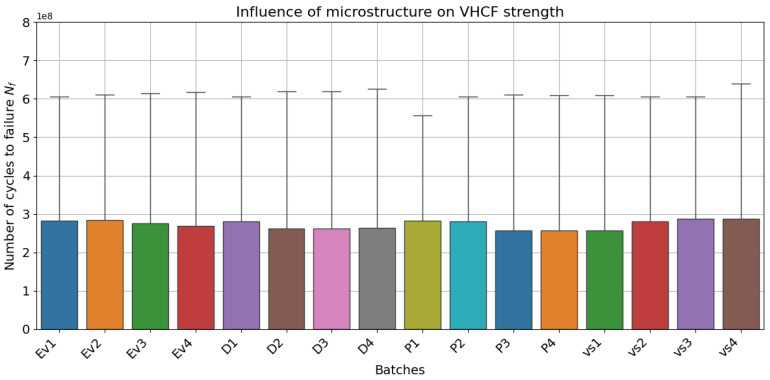
Box plot for average strength showing model sensitivity to microstructural features and evidence of complex interactions with features like porosity.

**Figure 8:**
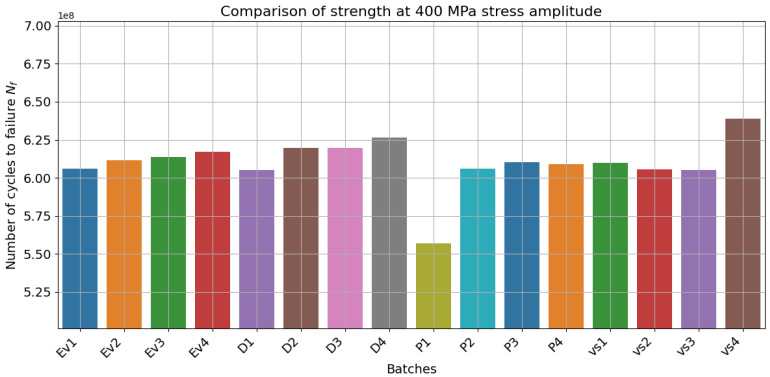
Comparison of strength at 400 MPa showing model sensitivity to microstructural features and evidence of complex interactions with features like porosity.

Figure 5 and Figure 6 show the model predictions for all investigated batches and their dependent parameters. In general, the model was able to infer the fatigue strength throughout the whole range, especially well in the VHCF range. Figure 7 and Figure 8 provide evidence of the sensitivity of the model to variations in the microstructure. The mean value of fatigue strength across all stress amplitude varies with every batch and its microstructural features. However, in Figure 8, it is evident that the fatigue strength follows a strength with energy density. Increased energy density in the second exposure led to improved fatigue life at 400 MPa of stress amplitude.

Several studies investigate the effect of loading frequency on fatigue properties, particularly in metallic materials and SLM-produced Ti-6Al-4V [7,16,46]. Research explores how pores, defects, and granular structures influence fatigue performance [47,48,49]. The usability of ultrasonic fatigue testing for high-cycle fatigue analysis is reviewed, with insights into AI-driven predictive models [44,50]. Studies integrate physics-informed machine learning and computational modeling for fatigue prediction and quality control in additive manufacturing [28,51,52]. The impact of processing parameters, alloy selection, and thermal treatments on fatigue behavior is explored [53,54,55]. Hong et al. [46] discuss the impact of loading frequency on fatigue behavior, highlighting that different frequencies can alter fatigue mechanisms due to changes in strain rates. This is particularly relevant for VHCF testing, where ultrasonic frequencies accelerate testing. Awd et al. [56] use numerical methods to study the influence of testing frequency on crack propagation in SLM Ti-6Al-4V, finding that higher loads lead to abrupt increases in fracture area before stabilizing.

The model was able to capture microstructural sensitivity to processing parameters, with fatigue performance varying according to grain morphology, phase distribution, and porosity. These findings are directly relevant to aerospace applications, where increased phase uniformity translates to improved fatigue life under very-high cyclic loading; to biomedical implants, where fine microstructural control reduces failure risk in load-bearing prosthetics; and to structural engineering, where porosity control enhances long-term durability in dynamically loaded structures.

## 4. Conclusions

This study highlights the significant advancements in understanding and predicting very high cycle fatigue (VHCF) behavior in additively manufactured materials, particularly Ti-6Al-4V. The model in Section 2.3 was able to capture and generate meaningful knowledge about the influence of microstructure on the complex phenomena of VHCF damage presented in Section 2.2. The finite element simulations based on the Fourier mapping in Equation (23) enabled estimation of state variables such as stress amplitudes and concentration at the nodal and defect scale as well as the macro scale of the homogenous elasto-plastic behavior. The distribution of the numerical results was used in augmenting the certainty of lifetime prediction in the maximum aposteriori estimation in Equation (37). This was a highly effective approach within specimens with low relative density and relatively enormous defect proportions. Through the integration of microstructural modeling, ultrasonic fatigue testing, and machine learning techniques, researchers have been able to enhance fatigue life predictions and optimize material properties. The validated model will be used to design microstructures that should deliver predefined fatigue performance limits, which are possible to manufacture through selective laser melting (SLM). Generative AI models will be built based on the results of this model, and the current model will be embedded in the foreseen pipeline. Key findings emphasize the role of loading frequency, microstructural characteristics, and process parameters in influencing fatigue performance. Additionally, the ML model could explain mechanistic phenomena such as the influence of frequency. The explainability of the results of machine learning models an enables an increase in robustness and trustworthiness in the decisions of AI models or its suggested process parameters in manufacturing and materials processing applications such as those in this study. In this context, the model generated physical knowledge by itself. It would be much more expensive to generate the same knowledge using classical experimental or exclusively physics-based numerical simulations. Moreover, the development of physics-informed machine learning frameworks offers a promising approach to reducing experimental costs while improving predictive accuracy. Future research should continue refining these computational models and explore, as mentioned above, generative AI models to further optimize fatigue-resistant structures and improve the reliability of additive manufacturing applications in aerospace, biomedical, and structural industries.

## Figures and Tables

**Table 1:** The process window used to train the model manufactured by the SLM of Ti-6Al-4V.

Parameter	Power	Spot Size	Scanning Velocity	Energy Density
Ti-6Al-4V Platform-Heated (PH) at 200 °C				
Batch	(P;W)	(vs; mm/s)	(D; mm)	(*Ev*; J/mm^3^)
Standard	240	1200	0.082	31.7
Ev1	200	1000	0.082	31.7
Ev2	160	800	0.082	31.7
Ev3	120	600	0.082	31.7
Ev4	80	400	0.082	31.7
P1	80	1200	0.082	10.6
P2	160	1200	0.082	21.2
P3	320	1200	0.082	42.3
P4	400	1200	0.082	52.9
vs1	240	3600	0.082	10.6
vs2	240	1800	0.082	21.2
vs3	240	900	0.082	42.3
vs4	240	720	0.082	52.9
D1	240	1200	0.116	31.7
D2	240	1200	0.142	31.7
D3	240	1200	0.164	31.7
D4	240	1200	0.183	31.7

No additional heat treatments are applied.

## Data Availability

The original contributions presented in this study are included in the article. Further inquiries can be directed to the corresponding author.

## Abbreviations

The following abbreviations are used in this manuscript:

AM	additive manufacturing
AI	Artificial Intelligence
BCC	Body-Centered Cubic
FEA	finite element analysis
HCP	Hexagonal Close-Packed
ID	internal damping
L-PBF	laser powder bed fusion
ML	machine learning
PDE	partial differential equation
PIML	physics-informed machine learning
PINN	physics-informed neural network
SLM	selective laser melting
USF	ultrasonic fatigue
VHCF	very high cycle fatigue
